# A patient with multiple primary malignant neoplasms with high variant allele frequencies of RB1, TP53, and TERT

**DOI:** 10.1186/s40364-024-00567-z

**Published:** 2024-02-06

**Authors:** Mingyang Ma, Kun Shang, Jiewei Wang, Xiaojing Teng, Peng Li, Jing Wang

**Affiliations:** 1 Department of Gastroenterology, Beijing Friendship Hospital, Capital Medical University, Beijing, China; 2 Department of Oncology, Beijing Friendship Hospital, Capital Medical University, Beijing, China; 3 Department of Pathology, Beijing Friendship Hospital, Capital Medical University, Beijing, China

**Keywords:** Multiple primary malignant neoplasms, Variant allele frequency, Next-generation sequencing, RB1, TP53, TERT, Small cell carcinoma of urinary bladder

## Abstract

Multiple primary malignant neoplasms are a rare disease with tumors of different histology or morphology arising in various sites. Next-generation sequencing is essential in the etiology, diagnosis, treatment, and surveillance of this disease. No eight primary malignant neoplasm cases with high variant allele frequencies of RB1, TP53, and TERT have been reported. Herein, we report a 65-year-old male who exhibited eight primary malignancies of the vocal cord, pharynx, kidney, mouth floor, esophagus, and urinary bladder with different pathological types. The first seven tumors were early-stage tumors; the last tumor, small cell carcinoma of urinary bladder, showed liver metastasis at diagnosis. Next-generation sequencing results revealed extremely high somatic variant allele frequencies of RB1 c.1472 T > C, TP53 c.576A > G, and TERT c.-58-u66C > T (95.5%, 95.1%, and 51.0%, respectively). No germline mutations were detected. These findings denoted a heavy tumor burden and poor prognosis. This is the first report of eight primary malignant neoplasm cases with high variant allele frequencies of RB1, TP53, and TERT.

**Supplementary Information:**

The online version contains supplementary material available at 10.1186/s40364-024-00567-z.

**To the editor,**

Multiple primary malignant neoplasms (MPMN) are tumors of different histology or morphology arising in various sites. With the progress in diagnostic methods, the prevalence of patients diagnosed with MPMN is increasing. Several reports have described cases of MPMN patients with two or more primary malignancies. However, only a few have presented cases from genetic sequencing. Here we describe a case of MPMN with eight tumors in the head and neck, esophagus, kidney, and bladder and a markedly high variant allele frequency in RB1, TP53, and TERT.

The patient was a single 65-year-old male who had smoked two packs of cigarettes and consumed 100 g of alcohol daily for the past 40 years. His father was diagnosed with esophageal carcinoma at the age of 55 and died of the disease at 60. His mother was diagnosed with breast cancer at the age of 50 and died of the disease at 52. His two brothers (59 and 62 years old when the study was performed) and one sister (55 years old) were healthy (Fig. 1).

**Fig. 1:**
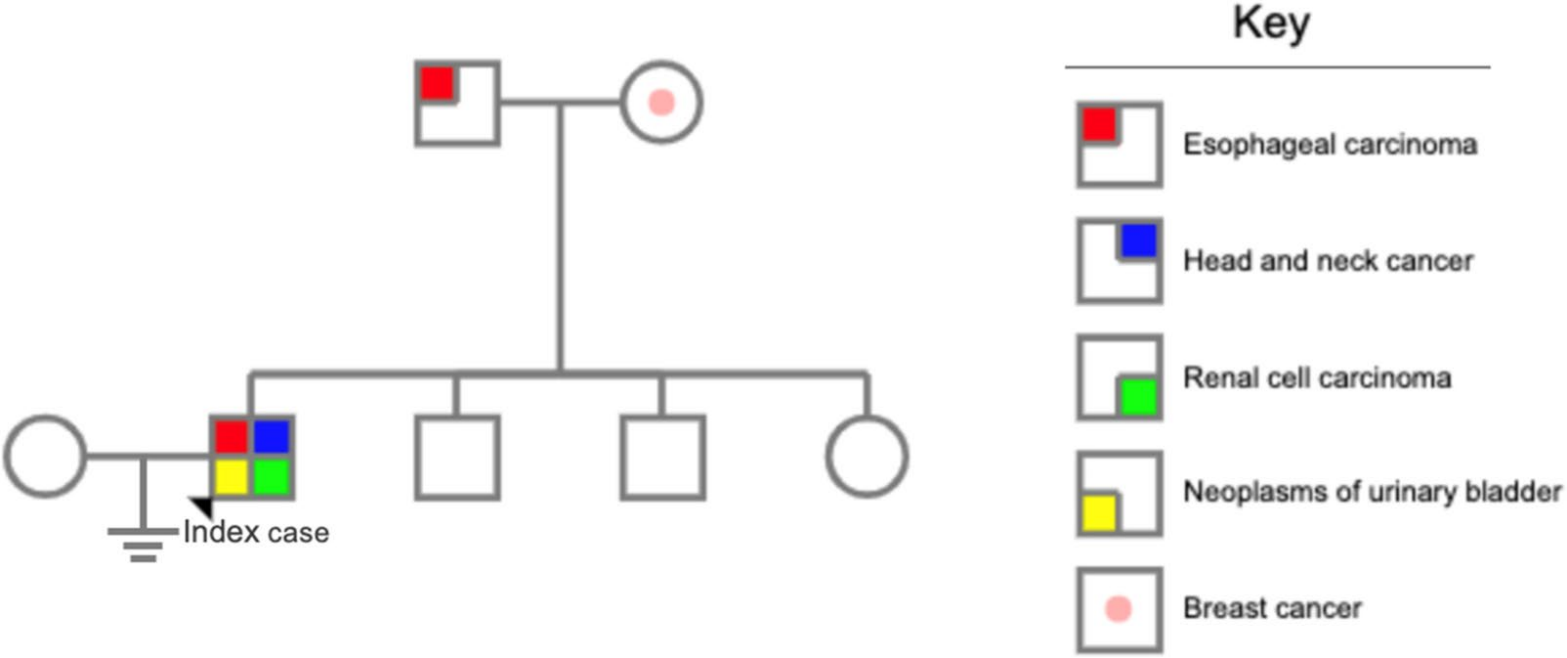
The pedigree of the patient’s family

From 57 to 65 years old, the patient was diagnosed with eight primary malignancies of the vocal cord, pharynx, kidney, mouth floor, esophagus, and urinary bladder with different pathological types (Table S1, Figs. S1, S2, S3, S4 and S5). The first seven tumors were identified and treated at the early stage; the last tumor, small cell carcinoma of urinary bladder (SCCB), showed liver metastasis after diagnosis (Fig. S6). Taking into account the patient’s history and family history of carcinoma, we performed DNA sequencing of the patient’s blood sample to determine the cause of the multiple primary malignant tumors. The variant allele frequencies (VAFs) of TP53, RB1, and TERT were extraordinarily high (95.5%, 95.1%, and 51.0%, respectively Table 1). There were no signs of germline mutations.

**Table 1:** Single nucleotide variant results of the patient

Gene variant		Reference sequence	VAF
RB1	c.1472 T > C	NM_000321.2	95.5%
TP53	c.578A > G	NM_000546.5	95.1%
TERT	c.-58-u66C > T	NM_198253.2	51.0%

Two definitions for multiple primary malignant tumors have been proposed. The Surveillance Epidemiology and End Results (SEER) program indicates that single tumors occurring in different parts of the same organ or tissue are deemed as single tumors [1]. In contrast, the Cancer Registries and International Agency for Research on Cancer (IACR/IARC) indicates that an organ with several tumors is considered one site [2]. In the U.S., most registries follow the SEER program guidelines because it includes the timing of diagnosis.

The incidence of MPMN among all cancers ranges from 2.4% to 17.2% [3]. The urinary bladder is the most common initial site in patients with multiple malignancies (16%), followed by the oral cavity and pharynx (15%) [4]. Federica et al. [5] found that the oral cavity and oropharynx were more common as index sites in the head and neck region (43% and 31%, respectively). Various factors play key roles in the development of multiple malignancies, such as genetics, hormones, prior cancer treatment exposure, and detrimental lifestyle factors (e.g., smoking and alcohol) [6, 7]. Upon searching the PubMed database, we identified seven cases of four or more primary tumors from 2010 to 2023 (summarized in Table S2). The literature search revealed that the current case is the only eight primary malignant neoplasm case reported with detailed DNA sequencing analysis.

Genetic sequencing analysis of the current patient revealed high VAFs of TP53, RB1, and TERT. High somatic mutations in TERT promoter regions are frequent in urothelial cancer [8]. Zheng et al. [9] examined patients with small cell carcinoma of different origins and found that the TERT C228T (c.-58-u66C > T) mutation was present in all SCCBs compared with small cell carcinoma of other origins. Notably, the case with SCCB had liver metastasis, suggesting that TERT promoter mutation may be a potential molecular marker to determine the primary site of SCCB. Wang et al. [10] found that RB1 expression, detected by immunohistochemistry, was absent in most SCCBs examined in their study. The authors concluded that inactivation of the RB1 gene may be involved in the oncogenesis of SCCB. TP53 mutation is associated with multiple human cancers, including bladder cancer [11]. The lack of CREBBP might increase the risk of this process via TP53 inactivation [12]. Whether RB1, TERT, and TP53 are driver mutations remains unclear. Chang et al. [8] discovered that TP53, RB, and TERT were most frequently altered genes in SCCB. However, urothelial bladder cancers also harbored these mutations, which may indicate that the mutations were essential but not sufficient for the development of small cell phenotype. These findings suggested a high tumor burden in the current patient, consistent with the diagnosis of SCCB with liver metastasis by CT, and indicated a bleak prognosis.

Given that the patient’s parents were diagnosed with cancer at 52 and 55 years old, we speculated that hereditary susceptibility might exist in this case. However, we were unable to obtain familial samples or further data as the patient’s parents had passed away and he had no descendants.

In conclusion, this is a rare case of eight primary malignant neoplasms. Next-generation sequencing identified an extremely high level of VAF in RB1, TP53, and TERT, denoting a heavy tumor burden and poor prognosis.

### Supplementary Information

**Additional file 1: Fig. S1.** Squamous cell carcinoma in the middle of the esophagus. The unstained area under endoscopy (A). En bloc resection of the lesion (B). **Fig. S2.** High-grade dysplasia of squamous epithelium on the left posterior pharyngeal wall. Intrapapillary capillary loop under NBI magnifying observation was classified as type B1 (A). En bloc resection of the lesion (B). **Fig. S3.** Two 1 cm isoechoic protrusions on the right wall of bladder (yellow arrows). **Fig. S4.** Ultrasound showed a hypoechoic mass in the posterior wall of the bladder (yellow arrow). **Fig. S5.** Hematoxylin-eosin staining (HE) and immunohistochemical staining (IHC) of some of the lesions. Vocal cord cancer (HE staining) (A); urothelium carcinomas of urinary bladder (HE staining) (B); urothelium carcinomas of urinary bladder (IHC with GATA antibody) (C); small cell carcinoma of urinary bladder (HE staining) (D); small cell carcinoma of urinary bladder (IHC with CgA antibody) (E); small cell carcinoma of urinary bladder (IHC with Syn antibody) (F). **Fig. S6.** CT image showed multiple liver metastases of SCCB.

**Additional file 2: Table S1.** Summary of the current case.

**Additional file 3: Table S2.** Cases of four or more primary tumors reported in PubMed from 2010 to 2023.

## Data Availability

All data generated or analyzed during this study are included in this published article.

## Abbreviations

MPMN: Multiple primary malignant neoplasm
SCCB: Small cell carcinoma of the bladder
VAF: Variant allele frequency
IACR/IARC: Cancer Registries and International Agency for Research on Cancer
SEER: Surveillance Epidemiology and End Results
IASLC: The International Association for the Study of Lung Cancer
