# Supplementary material for: A patient with multiple primary malignant neoplasms with high variant allele frequencies of RB1, TP53, and TERT
Source: Biomark Res. 2024 Feb 6;12:20. doi: 10.1186/s40364-024-00567-z (PMC10845515; doi:10.1186/s40364-024-00567-z)
Supplement: Supplementary file 1 — Additional file 1: Fig. S1. Squamous cell carcinoma in the middle of the esophagus. The unstained area under endoscopy (A). En bloc resection of the lesion (B). Fig. S2. High-grade dysplasia of squamous epithelium on the left posterior pharyngeal wall. Intrapapillary capillary loop under NBI magnifying observation was classified as type B1 (A). En bloc resection of the lesion (B). Fig. S3. Two 1 cm isoechoic protrusions on the right wall of bladder (yellow arrows). Fig. S4. Ultrasound showed a hypoechoic mass in the posterior wall of the bladder (yellow arrow). Fig. S5. Hematoxylin-eosin staining (HE) and immunohistochemical staining (IHC) of some of the lesions. Vocal cord cancer (HE staining) (A); urothelium carcinomas of urinary bladder (HE staining) (B); urothelium carcinomas of urinary bladder (IHC with GATA antibody) (C); small cell carcinoma of urinary bladder (HE staining) (D); small cell carcinoma of urinary bladder (IHC with CgA antibody) (E); small cell carcinoma of urinary bladder (IHC with Syn antibody) (F). Fig. S6. CT image showed multiple liver metastases of SCCB. [file 40364_2024_567_MOESM1_ESM.docx]

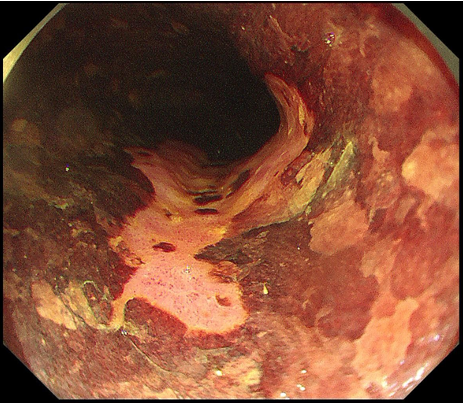

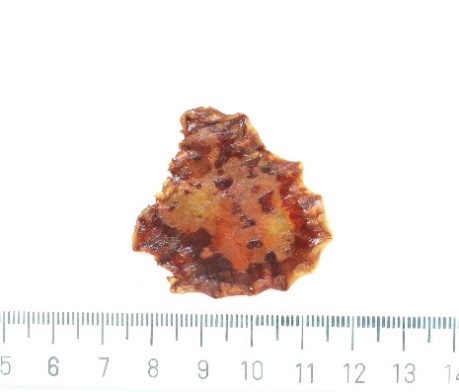


Fig. S1 Squamous cell carcinoma in the middle of the esophagus. The unstained area under endoscopy (A). En bloc resection of the lesion (B).


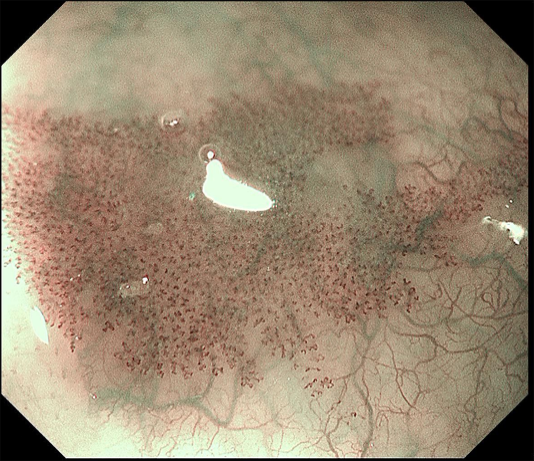

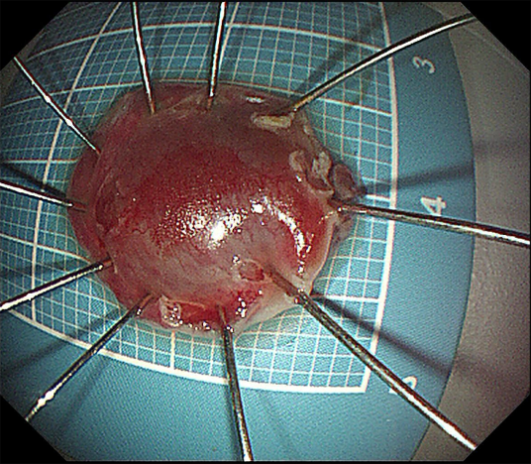


Fig. S2 High-grade dysplasia of squamous epithelium on the left posterior pharyngeal wall. Intrapapillary capillary loop under NBI magnifying observation was classified as type B1 (A). En bloc resection of the lesion (B)


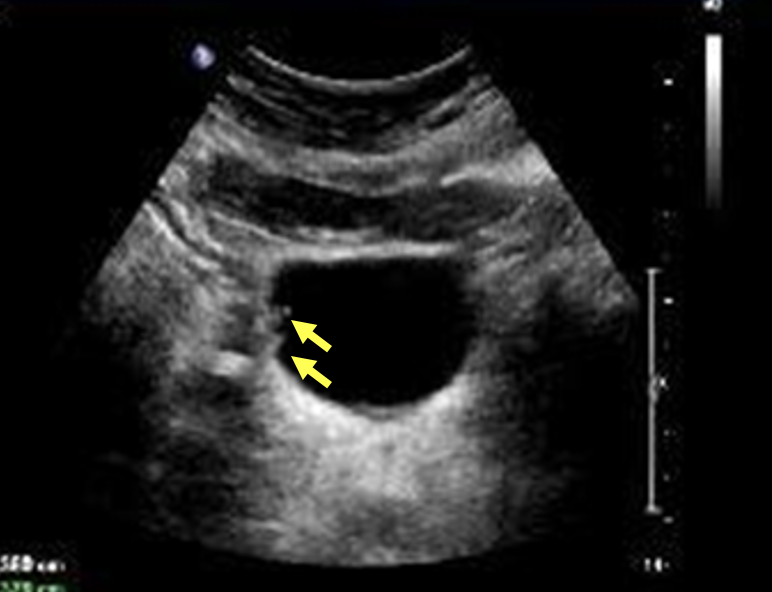


Fig. S3 Two 1 cm isoechoic protrusions on the right wall of bladder (yellow arrows).


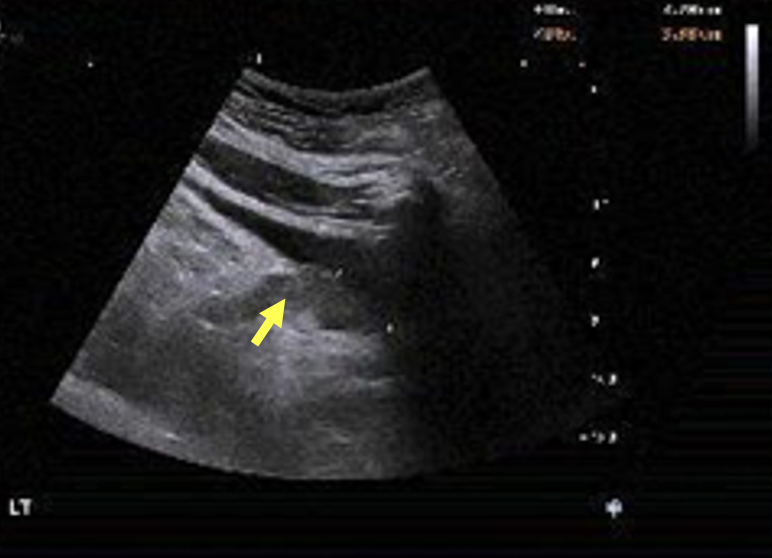


Fig. S4 Ultrasound showed a hypoechoic mass in the posterior wall of the bladder (yellow arrow).


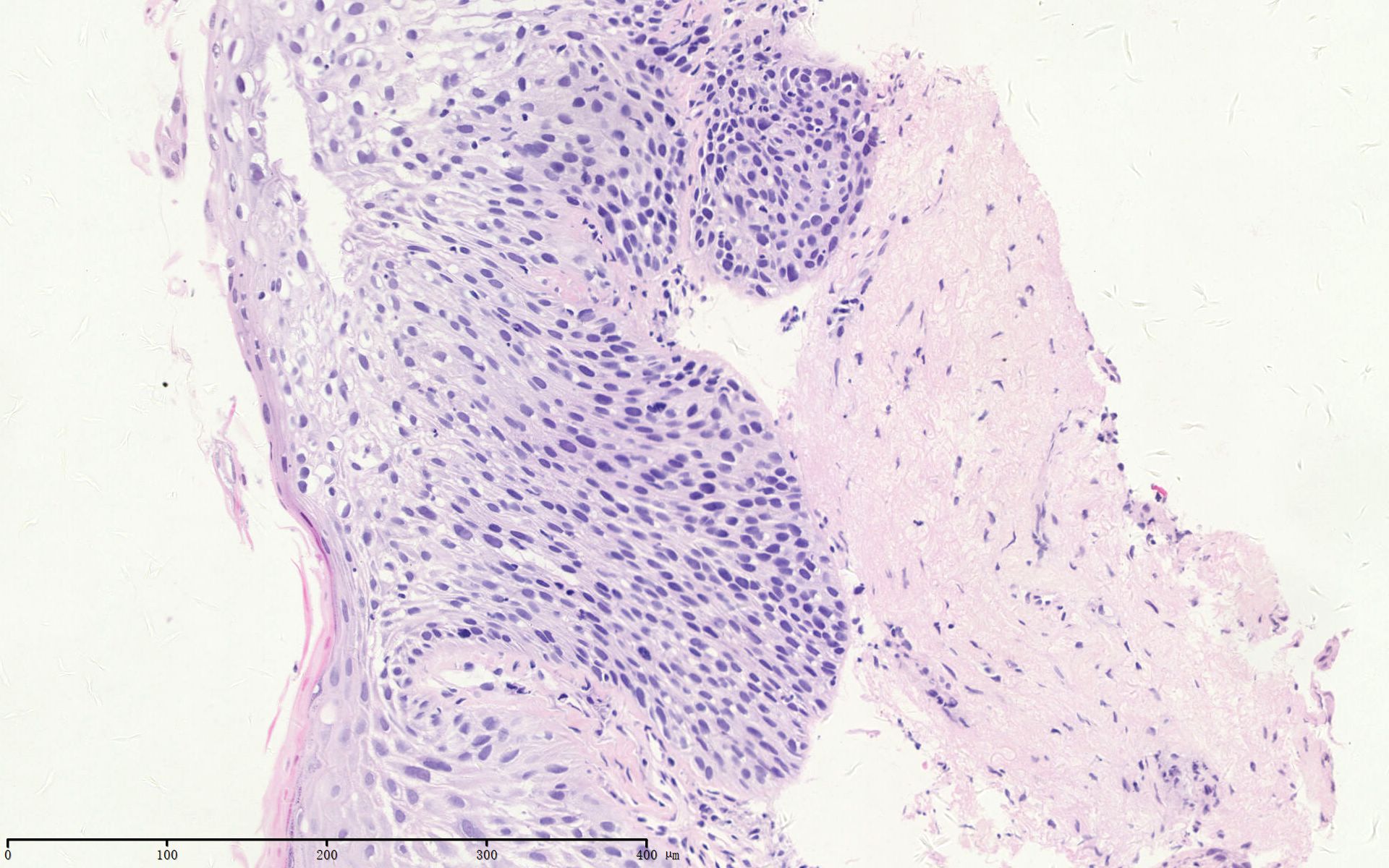


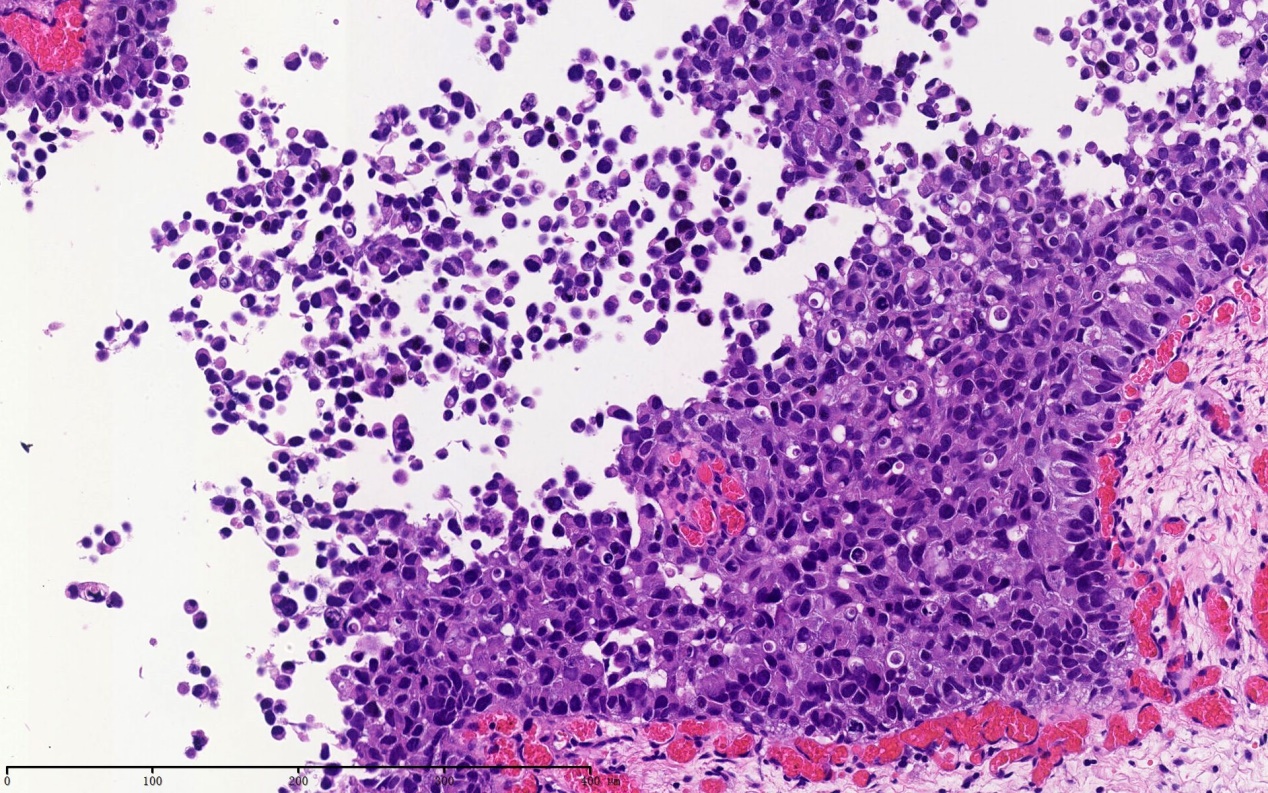


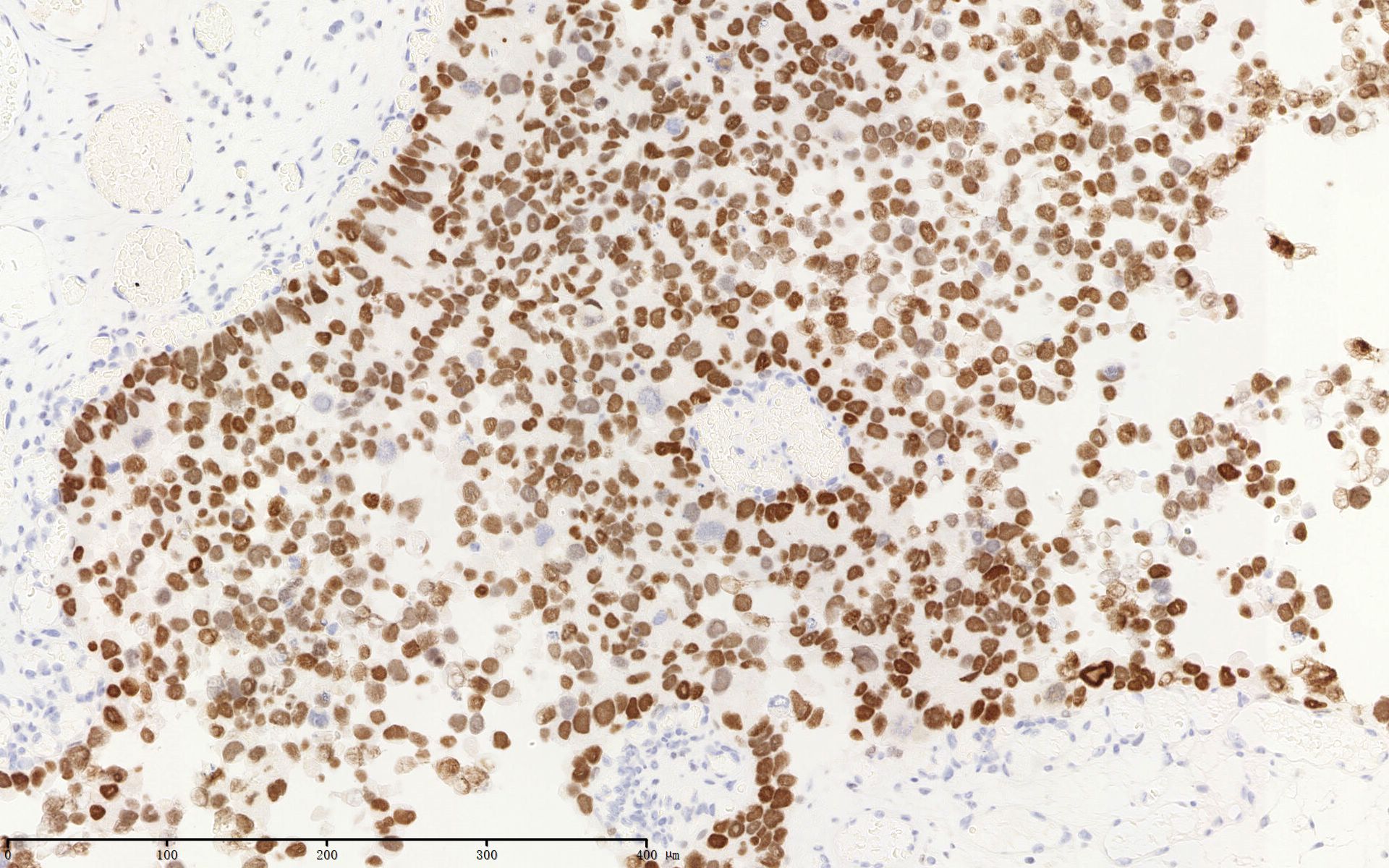


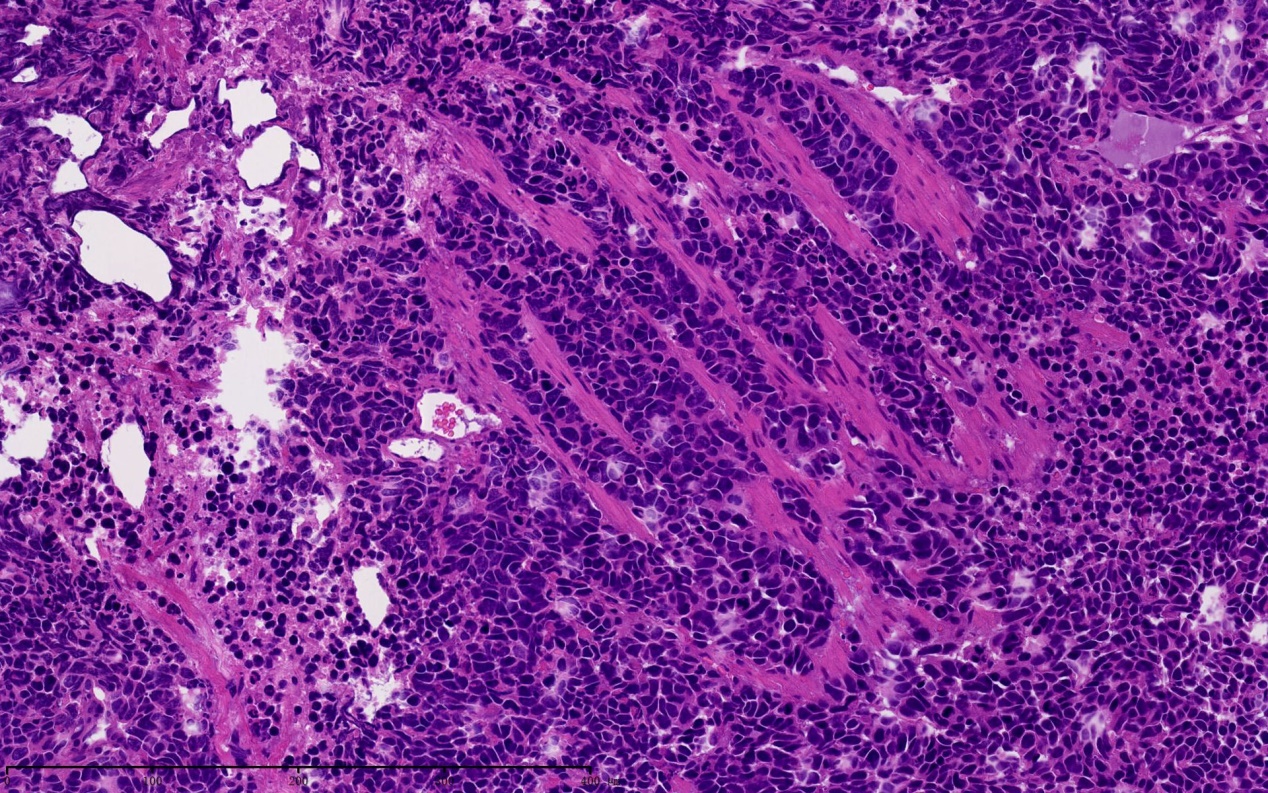


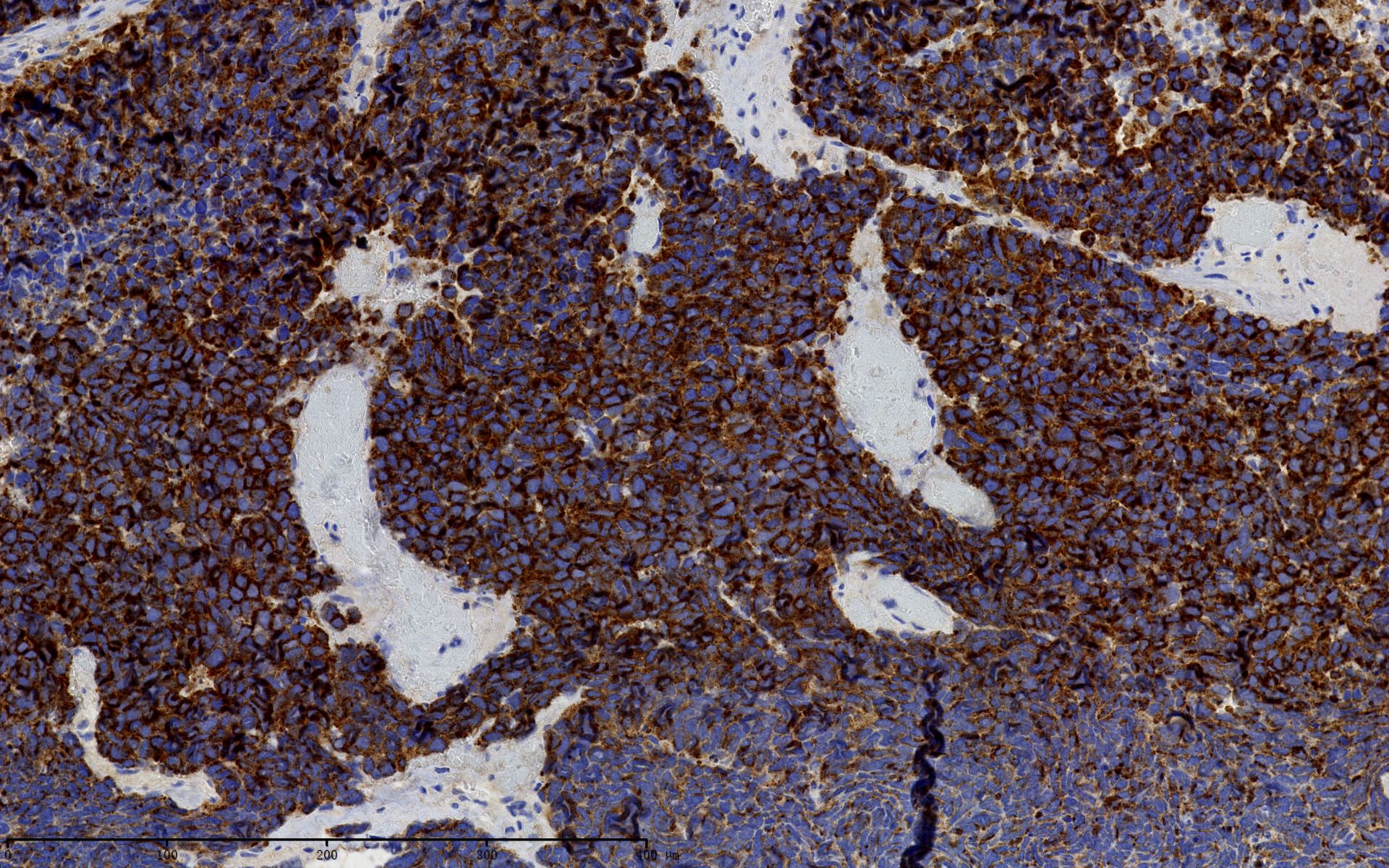


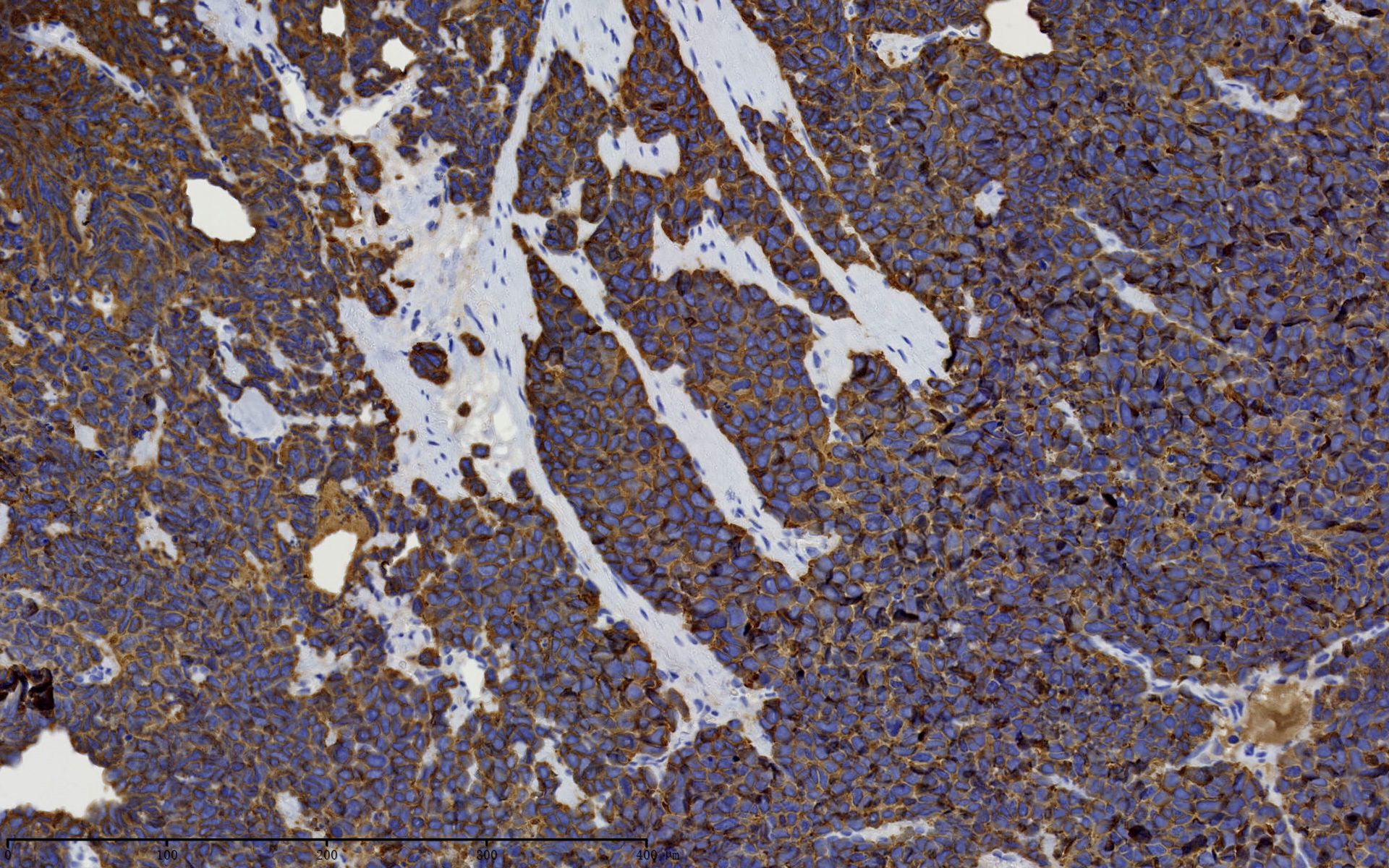


Fig. S5 Hematoxylin-eosin staining (HE) and immunohistochemical staining (IHC) of some of the lesions. Vocal cord cancer (HE staining) (A); urothelium carcinomas of urinary bladder (HE staining) (B); urothelium carcinomas of urinary bladder (IHC with GATA antibody) (C); small cell carcinoma of urinary bladder (HE staining) (D); small cell carcinoma of urinary bladder (IHC with CgA antibody) (E); small cell carcinoma of urinary bladder (IHC with Syn antibody) (F).


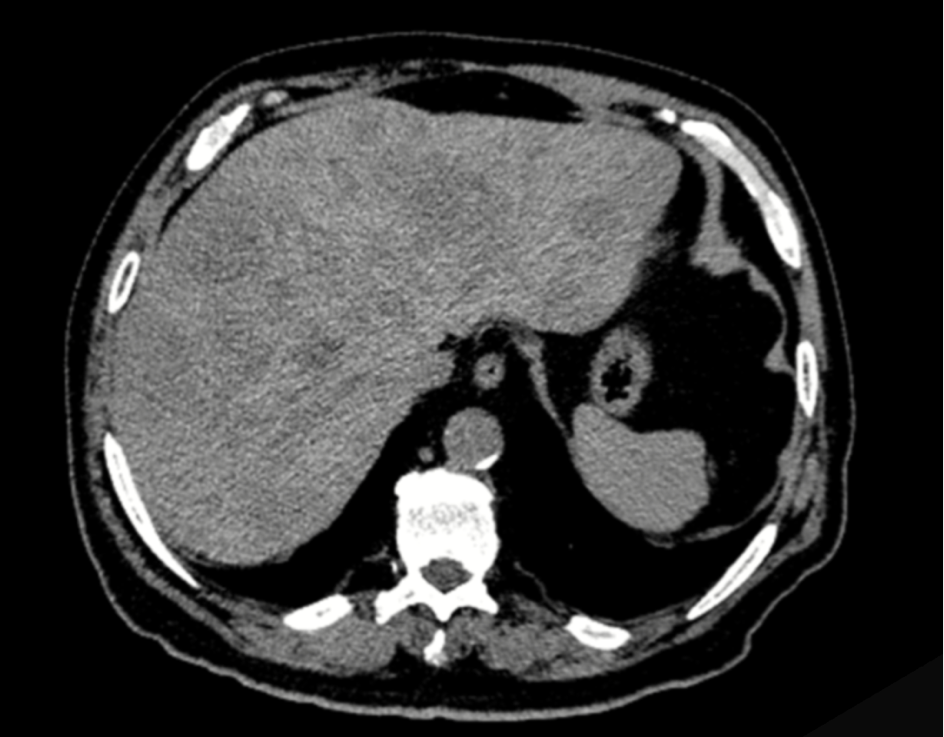


Fig. S6 CT image showed multiple liver metastases of SCCB
