# Supplementary material for: A patient with multiple primary malignant neoplasms with high variant allele frequencies of RB1, TP53, and TERT
Source: Biomark Res. 2024 Feb 6;12:20. doi: 10.1186/s40364-024-00567-z (PMC10845515; doi:10.1186/s40364-024-00567-z)
Supplement: Supplementary file 2 — Additional file 2: Table S1. Summary of the current case. [file 40364_2024_567_MOESM2_ESM.docx]

Table S1. Summary of the current case

| Tumor pathology and location | Age(years) | Immunohistology | Treatment | TNM 8^th^ edition classification |
| --- | --- | --- | --- | --- |
| Squamous cell carcinomas of vocal cord | 57 | - | surgery | Stage I (pT1N0M0) |
| Squamous cell carcinomas of pharyngeal | 57 | - | surgery | Stage I (pT1N0M0) |
| Adenocarcinoma of right upper kidney | 61 | CD10+, CD117-, CAIX+, EMA+, Vimentin+，E-cadherin-, CK7-, Pax-8+, P504s+, TFE-3+, CK+, SDHB-，FH+ | surgery | Stage I (pT1aN0M0) |
| Squamous cell carcinoma of mouth floor | 62 | - | surgery | Stage I (pTisN0M0) |
| Squamous carcinoma of esophagus | 63 | CD31+, CD34+, D2-40+, P53+, E-cadherin+, Desmin+, Ki67+ | ESD | Stage I (pT1aN0M0) |
| squamous carcinoma of pharyngeal | 63 | - | ESD | Stage I (pTisN0M0) |
| urothelium carcinomas of urinary bladder | 64 | GATA3+, CK7+, P63+, Her-2++, CD44V6+, EMA+, FGFR3-, P53+, Ki67+(30%) | TURBt | Stage I (pT1N0M0) |
| Small cell carcinoma of urinary bladder | 65 | GATA3-, CK7+, P63-, Her-2-, CD44V6-, EMA+, FGFR3-, P53+, Ki67+(70%), CD56+, CgA+, Syn+, CK5/6-, Uroplakin-3- | surgery | Stage IVB (pT3N2M1b) |

Age: age at diagnosis, ESD, endoscopic submucosal dissection; TURBt, trans-urethral resection of bladder tumor
