# Supplementary material for: A patient with multiple primary malignant neoplasms with high variant allele frequencies of RB1, TP53, and TERT
Source: Biomark Res. 2024 Feb 6;12:20. doi: 10.1186/s40364-024-00567-z (PMC10845515; doi:10.1186/s40364-024-00567-z)
Supplement: Supplementary file 3 — Additional file 3: Table S2. Cases of four or more primary tumors reported in PubMed from 2010 to 2023. [file 40364_2024_567_MOESM3_ESM.docx]

Table S2 Cases of four or more primary tumors reported in PubMed from 2010 to 2023

| **Author, year** | **Sex** | **Total tumor count** | **Tumors** | **Genetic test** | **Synchronous/metachronous** | **Onset age** | **Time span between the first and last tumors (years)** | **Survival (years)** | **Family history of cancer** |
| --- | --- | --- | --- | --- | --- | --- | --- | --- | --- |
| **Ahmed,2011(13)** | Female | 8 | Endometrial cancer/colorectal cancer/renal cell carcinoma /Endometrial cancer/breast cancer/lung cancer/transitional cell carcinoma of ureters/rhinocarcinoma | N/A | Synchronous and metachronous | 50 | 7 | Alive at the time of publication | N/A |
| **Yoshihiro(14), 2014** | Male | 7 | Squamous cell carcinoma (SCC) of the glottis, oral floor, and esophagus/urothelial carcinoma (UC) of the renal pelvis and urinary bladder/prostate cancer | N/A | Synchronous and metachronous | 56 | 2 | 2 | N/A |
| **Oana(15), 2015** | Female | 4 | Lung adenocarcinomas with different mutations | KRAS/EGFR | Synchronous | 72 | 0.17 | Alive at the time of publication | Her mother died of lung cancer |
| **Jenny(16), 2017** | Female | 5 | Endometrial cancer/colorectal cancer/malignant melanoma/breast cancer/pleomorphic spindle cell sarcoma | No mutation was found in analyzed genes | Synchronous and metachronous | 65 | 16 | Alive at the time of publication | Two sisters with breast cancer and a brother with lung cancer |
| **Wang(17), 2019** | Female | 5 | Endometrial cancer/transitional cell carcinoma of ureters and bladder/colorectal cancer/breast cancer | BRIP1/FNACG/NBN/AXIN2/SRD5A2/CEBPA | Metachronous | 50 | 19 | Alive at the time of publication | Her father and first older brother had gastric cancer; her second older brother was diagnosed with three primary cancers (colon cancer, squamous cell carcinoma of the skin and prostate cancer); her niece had leukemia. |
|  | Female | 4 | Endometrial cancer/ovarian serous cystadenocarcinoma/clear cell carcinoma of kidney/invasive ductal carcinoma breast cancer | BMPR1A/FANCD2/MLH3/BRCA2/FANCM | Synchronous and metachronous | 47 | 4 | Alive at the time of publication | Her father died of lung cancer |
| **Jia(18), 2021** | Male | 4 | Colorectal carcinomas/neuroendocrine tumor (NET) | KRAS/APC/PIK3CA/ERBB2 | Synchronous | 56 | 0 | Alive at the time of publication | N/A |
| **Wan(19), 2022** | Male | 4 | esophageal squamous cell carcinoma/urinary  tract urothelial carcinoma/small cell lung cancer/lung squamous cell carcinoma | N/A | Metachronous | 42 | 21 | Alive at the time of publication | N/A |

.

**References**

13. Slem A, Abu-Hijlih R, Abdelrahman F, Turfa R, Amarin R, Farah N, et al. Eight primary malignancies: case report and review of literature. Hematol Oncol Stem Cell Ther. 2011;4(4):185-7.

14. Mukaiyama Y, Suzuki M, Morikawa T, Mori Y, Takeshima Y, Fujimura T, et al. Multiple primary malignant neoplasms of the glottis, renal pelvis, urinary bladder, oral floor, prostate, and esophagus in a Japanese male patient: a case report. World J Surg Oncol. 2014;12:294.

15. Rafael OC, Lazzaro R, Hasanovic A. Molecular Testing in Multiple Synchronous Lung Adenocarcinomas: Case Report and Literature Review. Int J Surg Pathol. 2016;24(1):43-6.

16. Nyqvist J, Persson F, Parris TZ, Helou K, Kenne Sarenmalm E, Einbeigi Z, et al. Metachronous and Synchronous Occurrence of 5 Primary Malignancies in a Female Patient between 1997 and 2013: A Case Report with Germline and Somatic Genetic Analysis. Case Rep Oncol. 2017;10(3):1006-12.

17. Wang L, Wang H, Wang T, Liu J, Chen W, Wang Y, et al. Analysis of polymorphisms in genes associated with the FA/BRCA pathway in three patients with multiple primary malignant neoplasms. Artif Cells Nanomed Biotechnol. 2019;47(1):1101-12.

18. Jia X, Peng X, Sun J, Zhang T, Lin H, Bai T, et al. Genomic profiling of a patient with quadruple synchronous colorectal cancer: a case report. BMC Gastroenterol. 2021;21(1):360.

19. Wan L, Yin F-Y, Tan H-H, Meng L, Hu J-H, Xiao B-R, et al. Case report: Quadruple primary malignant neoplasms including esophageal, ureteral, and lung in an elderly male. Open Life Sci. 2022;17(1):1223-8.
